# Assessment of radiographic healing after tibial plateau leveling osteotomy in young and older dogs using the modified RUST scoring system

**DOI:** 10.3389/fvets.2026.1934451

**Published:** 2026-10-06

**Authors:** Bridget L. Anderson, Steven W. Frederick, Catherine Popovitch

**Affiliations:** 1 BluePearl Pet Hospital, Levittown, PA, United States; 2 BluePearl Science, Tampa, FL, United States

**Keywords:** age, dog, osteogenesis, radiographic osteotomy healing, tibial plateau leveling osteotomy

## Abstract

**Objective:**

To compare radiographic osteotomy healing scores after tibial plateau leveling osteotomy (TPLO) in young (2–5 years) and old (8–12 years) dogs using the modified Radiographic Union Score for Tibial fractures (mRUST) system, and to evaluate agreement between large language model-assisted interpretation (LLM), surgeon, and surgical resident scoring.

**Study design:**

Client-owned, 20–40 kg dogs treated with unilateral TPLO at ages 2–5 or 8–12 years between 2023 and 2025 were retrospectively identified. Dogs with endocrinopathy or atypical TPLO or recovery were excluded. Dogs were assigned to Group 1 (2–5 years) or Group 2 (8–12 years). 8-week postoperative radiographs were scored independently by an LLM, a surgeon, and a surgical resident using the 4-point mRUST system. Scores were compared between age groups using the Wilcoxon rank-sum test, and interrater agreement was assessed using an intraclass correlation coefficient (ICC).

**Results:**

Group 1 had 49 dogs; Group 2 had 51 dogs. Group 1 had a mean (SD) age of 3.82 (1.11) years, while Group 2 had a mean (SD) age of 9.24 (0.99) years. mRUST scores did not differ between age groups across LLM (*p* = 0.352), surgeon (*p* = 0.908), or resident assessments (*p* = 0.588). LLM, surgeon, and resident scores had only fair agreement (ICC = 0.4504, *p* < 0.001).

**Conclusion:**

Chronological age was not associated with mRUST score following TPLO at the 8-week postoperative evaluation. The mRUST system demonstrated moderate agreement between human observers. LLM scoring showed promise; further refinement is needed for clinical application.

## Introduction

Cranial cruciate ligament disease (CCLD) is a common cause of hindlimb lameness in dogs. The tibial plateau leveling osteotomy (TPLO) procedure has become a standard surgical intervention for CCL rupture (1). The procedure restores stability to the stifle joint by neutralizing cranial tibial thrust during the stance phase of gait by decreasing the tibial plateau angle (2, 3). However, it is important to note that post-procedure bone healing is a crucial determinant of success, as it affects the recovery time and the dog’s ability to return to full function.

Studies of humans have demonstrated that aging can be a risk factor for fractures due to decreased bone mass and bone quality (4). Additionally, differences in bone healing in mice and identified delayed chondrocyte differentiation and maturation, as well as impaired vascular invasion and bone formation in older animals compared to the younger group (5). This aligns with previous reports of delayed fracture healing in the elderly population, likely resulting from a lower capacity for mesenchymal stem cell differentiation and impaired angiogenesis/vasculogenesis (4). Despite the significance of age-related differences in bone healing identified in human medicine, limited studies have specifically compared radiographic bone healing between different age groups of dogs following TPLO surgery. Existing clinical literature has focused on clinical outcomes such as pain management, lameness resolution, gait analysis, osteoarthritis development, goniometry, and complication rates without specifically quantifying differences in bone healing based on age (6, 7). Therefore, a better understanding of how age influences bone healing outcomes is crucial for veterinarians to optimize postoperative care, tailor rehabilitation protocols, and provide accurate prognoses to pet owners.

Radiographic assessment of bone healing following TPLO plays a central role in postoperative clinical decision-making, including recommendations for activity restriction, progression of rehabilitation, and timing of follow-up evaluations. Although subjective interpretation of radiographs remains common in clinical practice, objective scoring systems have been increasingly adopted to standardize assessment. Various scales have been used to evaluate radiographic bone healing objectively, including variations on 5-, 10-, and 12-point scales (8–10). Each scoring system presents unique challenges, and currently, a single, standardized scoring system for evaluating osteotomy healing has not been established in veterinary medicine (11). The modified Radiographic Union Score for Tibial Fractures (mRUST), previously developed for human orthopedic medicine (12), has been adapted for veterinary applications to quantitatively assess cortical healing based on the presence of callus or a persistent fracture line (8).

The objective of this study was to evaluate the association between chronologic age and radiographic osteotomy healing following TPLO using the mRUST scoring system, with the aim of determining whether age alone is a reliable predictor of delayed radiographic union at a standardized postoperative time point. Our null hypothesis was that radiographic bone healing scores at 8 weeks postoperatively would not differ between younger (2–5 years) and older (8–12 years) dogs undergoing TPLO.

## Methods

### Study design and case selection

This retrospective cohort study evaluated radiographic osteotomy healing following TPLO in dogs treated at three private practice referral hospitals in Philadelphia, Pennsylvania, USA. Electronic medical records from three private practice referral hospital settings in Philadelphia, Pennsylvania, USA were retrospectively reviewed to identify dogs weighing 20–40 kg that underwent unilateral TPLO between January 2023 and January 2025. Dogs were categorized into two predefined age groups based on chronologic age at the time of surgery: Group 1 included dogs aged 2–5 years and Group 2 included dogs aged 8–12 years. Dogs aged 6–7 years were excluded to increase separation between the age groups for evaluation of a potential age-associated difference in radiographic osteotomy healing. A minimum of 45 dogs per group was estimated to provide 80% power at a two-sided α of 0.05 to detect a between-group difference in total mRUST score of 1.5 points, assuming a standard deviation of ≤2.5 points.

Dogs were included if they had a routine unilateral TPLO, as described by Slocum, when they were between the ages of 2–5 years (Group 1) or 8–12 years (Group 2) without any major complications on physical or radiographic examination during the first 8 postoperative weeks (2, 13). Furthermore, inclusion criteria involved complete medical records and available orthogonal stifle radiographs. Dogs were excluded if they were outside the predefined age ranges for Groups 1 and 2 at the time of surgery; had evidence of systemic disease based on hematologic and/or serum biochemical evaluation; experienced a major complication during or after surgery, as defined by Cook et al. (13); lacked complete medical records or postoperative radiographs; were receiving concurrent corticosteroid therapy; or had concurrent orthopedic or neurologic conditions identified at the time of surgery (e.g., medial patellar luxation, neuromuscular disease, or tendinopathy).

### Surgical procedure

Anesthetic protocols were not standardized across cases or participating institutions. Premedication, induction, maintenance, and perioperative analgesic protocols were selected by the anesthetist based on individual patient factors, including age, physical status, concurrent medical considerations, and preference. Therefore, anesthetic protocols differed between younger and older dogs. Specific anesthetic protocols were not evaluated as variables in the present study.

All TPLO procedures were performed by either a board-certified veterinary surgeon or an American College of Veterinary Surgeons resident under the direct supervision of a board-certified surgeon. Parapatellar mini-arthrotomies were performed in all dogs to confirm the diagnosis of CCL rupture. All CCLs were debrided at time of arthrotomy. If meniscal pathology was present at the time of surgery, a caudomedial hemimeniscectomy was performed. A curvilinear osteotomy was performed routinely using an oscillating radial blade. Internal fixation of the osteotomy was performed using locking TPLO plates with two different implant systems (DePuy Synthes Vet, Paoli, PA, and Arthrex Vet Systems, Naples, FL). Each dog who received a Synthes implant had two cortical screws placed distal to the osteotomy, with the remaining holes filled with locking screws. For each dog that received the Arthrex implant, one cortical screw was placed distal to the osteotomy, with the remaining holes filled with locking screws. All osteotomies were compressed using the compression hole with each TPLO plate. Each dog received appropriately sized implants, at the surgeon’s discretion, at the time of surgery. Routine closure was performed following all screw placements.

Postoperative orthogonal radiographs of the tibia to include the stifle proximally and tarsus distally were obtained immediately following surgery and at approximately 8 postoperative weeks. Confirmation of appropriate implant positioning, sizing, and sufficient tibial plateau angle modification was assessed for each radiograph to complete the case. Dogs recovered with the surgical service and were discharged with instructions regarding postoperative incision care, activity restrictions, and medication. All dogs in the study were discharged with an antibiotic medication (cefpodoxime 5–10 mg/kg PO q 24 h x 7–14 days or cephalexin 22 mg/kg PO q 12 h x 7–14 days), a non-steroidal anti-inflammatory medication (carprofen 2.2 mg/kg PO q 12 h), additional pain medication (gabapentin 10 mg/kg PO q 8 h), and a sedative (trazodone 5 mg/kg PO q 8 h). The duration of gabapentin and trazodone administration was not standardized across participating institutions and was individualized according to patient comfort, behavior, activity restrictions needed, clinician discretion, and owner-reported concerns. Similarly, standardized criteria for medication refills were not established, and additional gabapentin or trazodone was prescribed when deemed clinically appropriate based on individual patient needs.

Follow-up rechecks were performed through the surgical department at 2 postoperative weeks (via a virtual recheck for incision evaluation and gait analysis) and 8 postoperative weeks for physical and radiographic examinations. If concerns were noted via virtual recheck or if owner concerns were reported, in-person rechecks were performed for further assessment. Owners were given postoperative activity instructions following completion of the initial 2-week recheck, which included increasing slow, controlled leash walks up to 10 min daily, with increments of 5 min weekly, until the in-person 8-week recheck. Refills of sedative or pain medications were provided upon request.

### Radiographic evaluation

All 8-week follow-up radiographs were collected for evaluation by a small animal surgical resident (BA), a board-certified small animal surgeon (CP), and an LLM assessment method. The board-certified surgeon was provided only anonymized radiographs and was blinded to patient identity, clinical information, chronological age, and age group. The surgical resident was responsible for case identification, data collection, and radiograph collection and therefore had access to patient identifying and clinical information; however, patient age and age group were not referenced at the time of radiographic scoring. The surgeon and surgical resident performed their assessments independently and were blinded to each other’s mRUST scores. The LLM was not provided with patient identity, clinical information, chronological age, age group, or scores assigned by human raters.

Osteotomy site healing was scored using the mRUST scoring system. Four cortices were independently evaluated on orthogonal radiographic projections: the cranial and caudal cortices on the mediolateral projection and the medial and lateral cortices on the craniocaudal projection. Each cortex was assigned a score from 1 to 4 according to the degree of radiographic healing: 1 = no callus formation with a clearly visible osteotomy line; 2 = callus formation with a persistent visible osteotomy line; 3 = bridging callus with an indistinct or absent osteotomy line; 4 = complete cortical remodeling with restoration of cortical continuity and no visible osteotomy line. Scores from the four cortices were summed to generate a total mRUST score ranging from 4 to 16, with higher scores representing greater radiographic osteotomy healing. The same scoring criteria were used by all three assessment methods: a small animal surgical resident, a board-certified surgeon, and LLM-assisted interpretation.

All human-assessed images were evaluated using a single PACS program (Keystone Asteris, Montreal, Canada). For large-language model-assisted assessment, postoperative orthogonal TPLO radiographs were uploaded to ChatGPT (OpenAI, San Francisco, CA, USA) in June 2025. The specific underlying model version available through the ChatGPT interface at the time of assessment was not recorded. Multiple dogs were evaluated sequentially within the same conversation. The same standardized instructions and mRUST scoring criteria were provided for each case. Prior to scoring, the model was instructed to review the predefined mRUST criteria and subsequently assign an individual score from 1 to 4 to each of the cranial, caudal, medial, and lateral cortices based on corresponding orthogonal radiographic views (Figure 1). The four cortical scores were recorded and summed to generate a total mRUST score ranging from 4 to 16. The same prompt was used for evaluating each radiograph and has been provided in Supplementary material 1.

**Figure 1:**
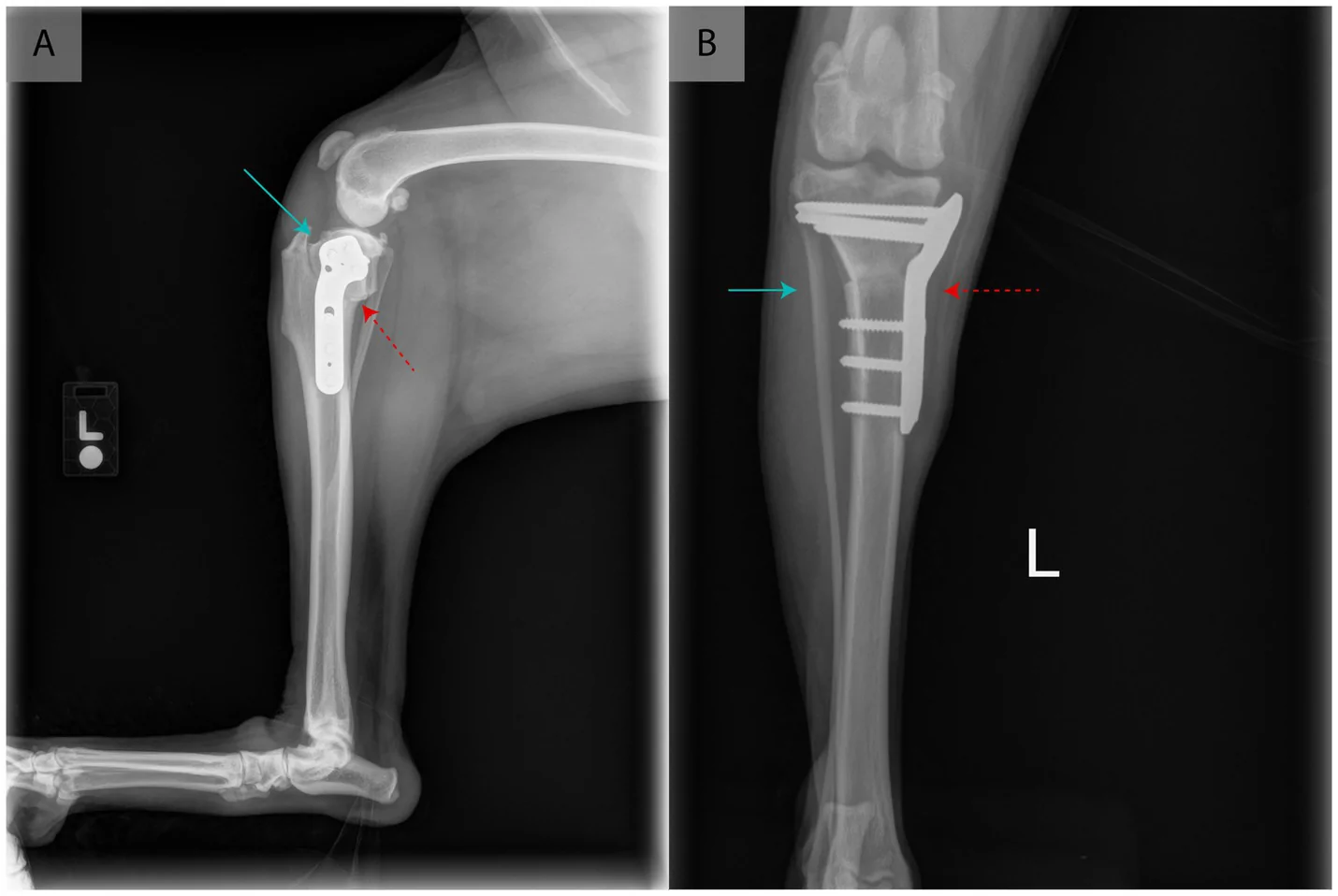
Representative orthogonal radiographs obtained approximately 8 weeks following tibial plateau leveling osteotomy (TPLO), demonstrating the four cortices evaluated using the modified Radiographic Union Score for Tibia fractures (mRUST). The cranial (blue arrow) and caudal (red dotted arrow) cortices are identified on the mediolateral projection **(A)**, and the medial (red dotted arrow) and lateral (blue arrow) cortices are identified on the craniocaudal projection **(B)**. Each cortex was assigned an mRUST score from 1 to 4 based on the degree of radiographic osteotomy healing and the four cortical scores were summed to generate a total mRUST score ranging from 4 to 16.

### Statistical analysis

Continuous numerical variables were assessed for normality with the Shapiro–Wilk test. Parametrically distributed numerical variables were summarized as mean ± standard deviation (SD), and ordinal and nonparametrically distributed numerical variables are summarized with median and interquartile range (IQR). Parametrically distributed numerical variables were compared using the Student’s *t*-test, and nonparametrically distributed numerical variables were compared with the Wilcoxon rank sum test. Frequency distributions between categorical variables were compared with the chi-square test of independence. Total mRUST scores were compared between age groups for each assessment method using the Wilcoxon rank-sum test. Statistical significance was set at *α* = 0.05.

Interrater agreement for total mRUST scores among the LLM, surgeon, and resident was assessed using a single-measure, absolute agreement intraclass correlation coefficient (ICC) based on a one-way random-effects model, with 95% confidence intervals. Agreement between surgeon and surgical resident total mRUST scores was also assessed separately using the same method.

## Results

A total of 439 dogs were initially identified. Eighty-seven (19.8%) were excluded due to loss of follow-up, 130 (29.6%) did not meet the age or weight criteria for enrollment, 43 (9.8%) underwent simultaneous bilateral TPLO procedures, and 79 (18%) had previously undergone TPLO on the contralateral limb. The remaining 100 dogs (22.8%) met all inclusion criteria, with 49 dogs assigned to Group 1 and 51 dogs assigned to Group 2. Group 1 included 29 (59.2%) spayed females and 20 (40.8%) neutered males, while Group 2 included 26 (51.0%) spayed females, 23 (45.1%) neutered males, and 2 (3.9%) intact males. Mixed-breed dogs were the most common in both groups (51.0% in Group 1 and 60.8% in Group 2), followed by Labrador Retrievers, Pitbulls, and Golden Retrievers. The median interval between surgery and follow-up radiographs was 56 days (range, 54–65 days).

Age differed due to study design; Group 1 had a mean (SD) age of 3.82 (1.11) years, while Group 2 had a mean (SD) age of 9.24 (0.99) years. Group 1 dogs were slightly heavier than Group 2 dogs (median weight 32.6 [IQR: 29.0–34.6] kg and 29.9 [IQR: 24.0–34.3] kg, respectively; *p* = 0.007). Sex distribution did not significantly differ between groups (*p* = 0.311). Implant type differed significantly between groups: 75.5% of dogs in Group 1 received Arthrex implants, compared to 54.9% in Group 2 (*p* = 0.031). The majority (58.8%) of dogs in group 2 underwent meniscectomy, compared to 34.7% in Group 1 (*p* = 0.016).

LLM-assessed, surgeon-assessed, and resident-assessed total mRUST scores did not significantly differ between age groups (*p* = 0.352, *p* = 0.908, and *p* = 0.588, respectively; Table 1; Figure 2). Median, interquartile range, and range of mRUST scores for each assessment method and age group are summarized in Table 1.

**Table 1:** Comparison of modified Radiographic Union Scores for Tibial fractures (mRUST) scores between age groups by assessment method.

Assessment method	Group 1 (*n* = 49)		Group 2 (*n* = 51)		*p*-value^*^
	Median (IQR)	Range	Median (IQR)	Range	
LLM	12 (11–12)	8–13	12 (11–12)	8–14	0.352
Surgeon	12 (10–13)	8–15	11 (10–13)	8–16	0.908
Resident	11 (10–13)	8–16	11 (10–12)	9–16	0.588

Data are presented as median (interquartile range [IQR]) and range. Group 1 included dogs 2–5 years of age (*n* = 49), and Group 2 included dogs 8–12 years of age (*n* = 51). LLM, large language model; mRUST, modified Radiographic Union Score for Tibial fractures. *p*-values represent comparisons of total mRUST scores between age groups using the Wilcoxon rank-sum test.

*p-value comparing same assessment method scores for age group 1 to scores for age group 2 dogs.

**Figure 2:**
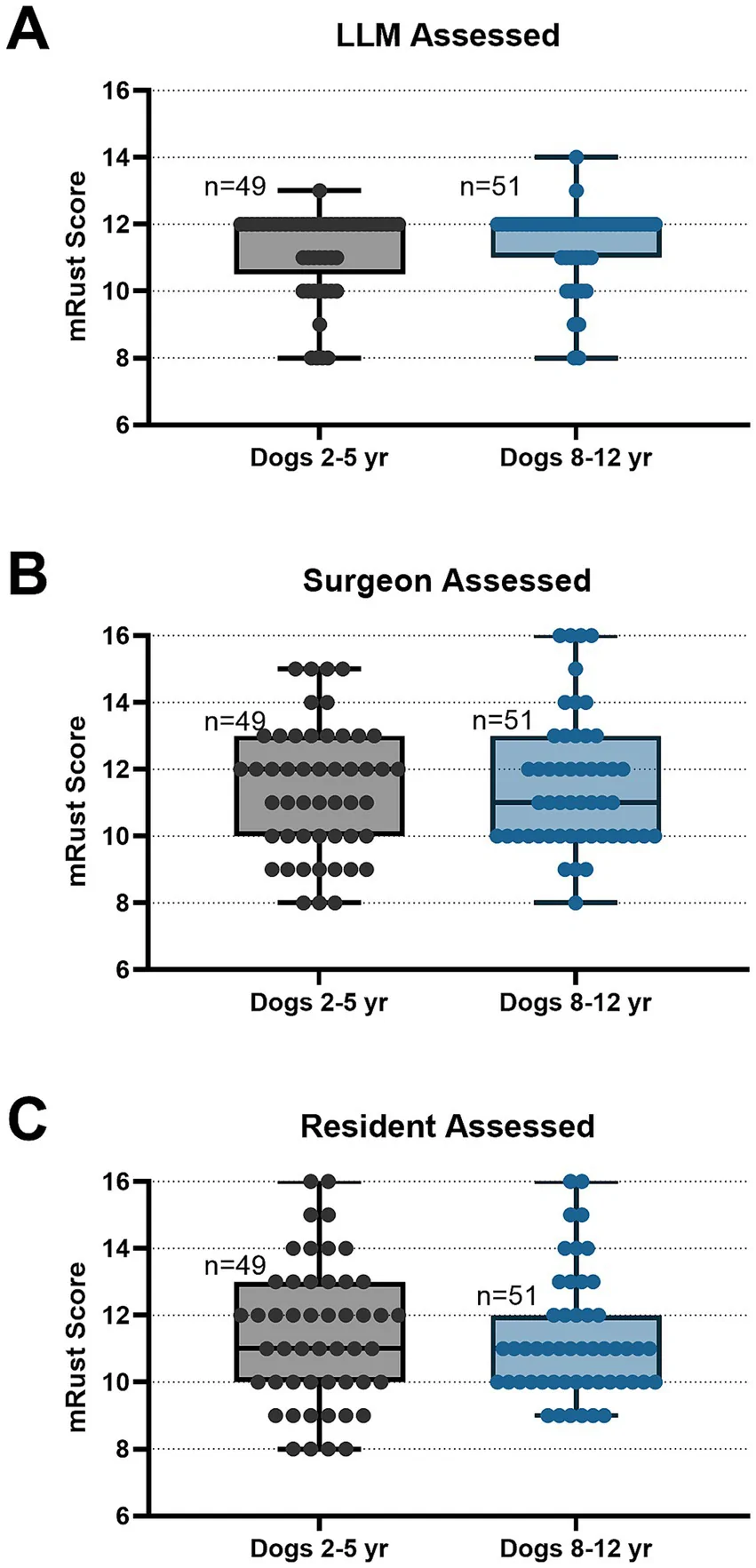
Distribution of total modified Radiographic Union Score for Tibial fractures (mRUST) scores between younger (Group 1; 2–5 years, *n* = 49) and older (Group 2; 8–12 years, *n* = 51) dogs approximately 8 weeks following tibial plateau leveling osteotomy. Individual mRUST scores are shown for **(A)** LLM assessment, **(B)** board-certified surgeon assessment, and **(C)** surgical resident assessment. No significant differences in mRUST scores were identified between age groups for the LLM-assisted (*p* = 0.352), surgeon (*p* = 0.908), or resident (*p* = 0.588) assessments.

LLM-assessed mRUST scores, surgeon-assessed scores, and resident-assessed scores demonstrated fair interrater agreement [single-measure, absolute agreement (AA) ICC = 0.4504 (95% CI: 0.3305–0.5664; *p* < 0.001)]. Agreement between surgeon-assessed scores and resident-assessed scores was stronger [single-measure, AA ICC = 0.7923 (95% CI: 0.7065–0.8552; *p* < 0.001)].

## Discussion

This retrospective cohort study found no statistically significant difference in 8-week radiographic osteotomy healing scores between younger and older adult dogs undergoing TPLO using the mRUST scoring system These findings suggest that chronological age was not associated with radiographic osteotomy healing at the evaluated postoperative time point; however, differences in the earlier rate or progression of healing cannot be excluded. The null hypothesis could not be rejected, as no significant differences in radiographic healing were identified between younger and older dogs at 8 weeks following TPLO.

Prospective studies evaluating TPLO radiographic bone healing at frequent intervals over 2–12 postoperative weeks have consistently found that routine healing with no additional intervention occurs within 8–12 weeks (14, 15). However, none of those studies evaluated the impact of age on bone healing, despite Franklin et al. positing that slightly different bone healing scores between groups at 7 and 10 postoperative weeks was likely attributed more to differences in dog age between their groups rather than the use of platelet rich plasma (15). In our study, radiographic osteotomy healing was evaluated at approximately 8 weeks postoperative because this represents the routine follow-up protocol at contributing hospitals. The timing of this evaluation was relatively consistent across the study population, with follow-up radiographs obtained at a median of 56 days postoperatively (range, 54–65 days). Although this time point is clinically relevant for postoperative decision-making (11), evaluation at a single interval limits assessment of the progression of osteotomy healing. It is possible that age-related differences may have been present earlier in the healing process but were no longer detectable by 8-weeks. For example, younger dogs may have demonstrated earlier onset or more rapid progression of osteogenesis, while older dogs reached similar radiographic healing scores by the 8-week evaluation. Therefore, the absence of detectable differences at 8-weeks should not be interpreted as evidence that the rate or pattern of osteotomy healing is equivalent between age groups. Prospective studies to include serial radiographic assessments are needed to determine whether chronological age influences the rate of radiographic osteotomy healing following TPLO.

The absence of a statistically significant difference between age groups should be interpreted cautiously. One possible explanation is that chronological age alone does not represent a clinically relevant determinant of radiographic osteotomy healing following TPLO at the evaluated postoperative time point of 8-weeks. This finding supports the continued use of standardized radiographic assessment to guide postoperative follow-up and client counseling, rather than modifying expectations based solely on patient age. From a clinical perspective, these findings suggest that chronological age along may not show expectation of delayed radiographic osteotomy healing at approximately 8 weeks following TPLO; however, small age-related differences cannot be excluded.

Alternatively, the lack of statistically significant differences may reflect limitations inherent to the study design rather than differences in healing based on age. The retrospective nature of this study, evaluation of a single postoperative time point, and variability in patient characteristics may have reduced the ability to detect subtle or delayed differences in healing. Although dogs were excluded if they had presence of other comorbidities that may affect healing, factors that were not evaluated in the current study such as bone quality or postoperative activity level that may have influenced radiographic healing were not available for analysis. These findings suggest that while chronologic age alone should not be assumed to predict delayed radiographic healing following TPLO, further prospective studies incorporating multiple postoperative time points and functional outcome measures are warranted to more fully characterize the relationship between age, radiographic healing, and clinical recovery.

An additional limitation is the use of chronological age to define the study groups. Biological aging varies among dogs and may be influenced by factors including breed, body size, genetics, and individual health status; therefore, dogs of the same chronological age may not be at equivalent stages of biological aging. Enrollment was restricted to dogs weighing 20–40 kg to reduce some of the variability associated with different body sizes. The predefined age ranges used in this study were intended to create separation between younger and older adult populations rather than represent standardized biological aging categories. Future studies incorporating additional measures of biological or physiologic aging may better characterize the relationship between aging and radiographic osteotomy healing.

A secondary aim of this study was to evaluate agreement between LLM-assisted and human mRUST assessment. LLM-assisted scores demonstrated a narrower distribution than scores assigned by the human observers, with 62% of dogs receiving a total mRUST score of 12 and 88% receiving scores between 10 and 12. Agreement among the three assessment methods was fair. Importantly, the present study was designed to evaluate agreement rather than diagnostic accuracy. No independent reference standard for radiographic osteotomy healing was available; therefore, disagreement between the LLM and human observers cannot establish which assessment was more accurate. Similarly, the narrower distribution of LLM-assisted scores should not necessarily be interpreted as greater consistency or superior performance, as this clustering may instead reflect reduced sensitivity to subtle differences in radiographic appearance. These findings support further investigation of LLM-assisted mRUST assessment using a validated reference standard before conclusions regarding diagnostic accuracy or clinical utility can be made. Agreement among all three assessment methods was fair, whereas agreement between the surgeon and resident was substantially greater.

Existing evidence across both human and veterinary musculoskeletal imaging highlights several key considerations when discussing the use of a computer-assisted scoring approach in radiographic interpretations. First, numerous studies report that large language model-assisted interpretation can achieve fracture-detection performance similar to that of trained clinicians, though this level of accuracy is not universal (16). Second, systematic review indicates that while large language models are generally effective, they continue to face challenges in identifying subtle radiographic abnormalities (17). Third, in veterinary medicine specifically, comparative studies show large language model-assisted interpretation may approach but not fully replace board-certified radiologists, and its role is more likely to augment rather than replace human interpretation (18). In the context of osteotomy healing scoring (such as our use of the mRUST system), the clustering of LLM-assisted scores may limit the ability to detect subtle differences in radiographic osteotomy healing or remodeling. Therefore, future work should aim for combined human-large-language model workflows, incorporate calibration of large language model-assisted interpretation output with functional/clinical outcomes (gait-analysis, force plate) and explore algorithmic training on such specific post-osteotomy healing datasets rather than primary fracture detection alone.

Several potential confounding variables differed between age groups, including implant system and frequency of caudomedial hemimeniscectomy. Implant type could theoretically influence radiographic osteotomy healing through differences in plate design and construct mechanics, which may alter stability and interfragmentary motion at the osteotomy site. Although both implant systems used in this study were locking TPLO plates and osteotomy compression was performed in both systems, differences in screw configuration and implant design were present. The study was not specifically designed to evaluate the effect of implant system on radiographic osteotomy healing; therefore, an effect of implant selection cannot be excluded. Meniscectomy was also performed more frequently in older dogs. Because caudomedial hemimeniscectomy was performed when meniscal pathology was identified at surgery, this difference may reflect variation in the severity or chronicity of underlying stifle disease between age groups. Meniscal pathology and its treatment could potentially influence postoperative joint inflammation, comfort, limb use, and loading of the operated limb, although a direct effect of meniscectomy on osteotomy healing has not been established in this study. Nevertheless, the unequal distribution of implant type and meniscectomy between age groups represents a potential source of confounding and should be considered when interpreting the relationship between chronological age and radiographic osteotomy healing. Additionally, although patient age and age group were not referenced or known to the surgical resident during radiographic assessment and the human observers scored the radiographs independently, the resident was involved in case identification and data collection and therefore was not completely blinded to other patient information. The potential for observer bias related to incomplete blinding cannot be entirely excluded. Radiographic quality, anesthetic management, perioperative and postoperative medication protocols, surgical technique, and rehabilitation protocols were also not standardized across all cases. The available sample size exceeded the estimated minimum of 45 dogs per group required to detect a 1.5-point difference in total mRUST score with 80% power. However, smaller differences between age groups may not have been detected and cannot be excluded. Moreover, retrospective evaluation of multi-institutional, real-world cases enhances external validity and clinical applicability. Finally, although radiographic osteotomy healing is a critical determinant in clinical decision-making, it may not fully reflect mechanical strength or return of function. Radiographs remain the most widely used tool for postoperative evaluation, but pairing standardized scoring with objective outcome measures should be a priority for future research.

This study found no statistically significant difference in 8-week radiographic osteotomy healing scores between younger and older dogs undergoing TPLO. Within the limitations of the present study, chronological age was not associated with mRUST score at the evaluated postoperative time point; however, smaller age-related differences or differences occurring earlier in the healing process cannot be excluded. The mRUST system demonstrated agreement between the two human observers was greater than the overall agreement observed among all three assessment methods. Because no independent reference standard was available, the diagnostic accuracy of LLM-assisted scoring could not be determined. These findings suggest that chronological age alone may not be a reliable predictor of radiographic osteotomy healing at approximately 8 weeks following TPLO and support the continued use of individual radiographic assessment when making postoperative recommendations. Further studies incorporating standardized LLM methodology, independent reference standards, and prospective validation are necessary before the clinical utility of LLM-assisted radiographic osteotomy scoring can be determined.

## Data Availability

The original contributions presented in the study are included in the article/Supplementary material, further inquiries can be directed to the corresponding author.
