# Supplementary material for: Assessment of radiographic healing after tibial plateau leveling osteotomy in young and older dogs using the modified RUST scoring system
Source: Front Vet Sci. 2026 Oct 6;13:1934451. doi: 10.3389/fvets.2026.1934451 (PMC13680158; doi:10.3389/fvets.2026.1934451)
Supplement: Supplementary file 1 [file Supplementary_file_1.docx]

**Supplementary Material 1.** Standardized prompt used for LLM-assisted mRUST assessment: You will be provided with orthogonal postoperative radiographs from a dog following tibial plateau leveling osteotomy (TPLO). Evaluate radiographic osteotomy healing using the modified Radiographic Union Score for Tibial fractures (mRUST). Evaluate each of the four cortices individually: cranial cortex on the mediolateral projection, caudal cortex on the mediolateral projection, medial cortex on the craniocaudal projection, and lateral cortex on the craniocaudal projection. Assign each cortex a score from 1 to 4 using the predefined criteria: 1 – no callus formation with visible osteotomy line; 2 – callus formation with a persistent visible osteotomy line; 3 – bridging callus with an indistinct or absent osteotomy line; 4 – complete cortical remodeling with restoration of cortical continuity and no visible osteotomy line. For each set of uploaded radiographs, provide an individual mRUST score for the cranial, caudal, medial, and lateral cortices. Then sum the four cortical scores to provide a total mRUST score.
